# Computational analysis of envelope glycoproteins from diverse geographical isolates of bovine leukemia virus identifies highly conserved peptide motifs

**DOI:** 10.1186/s12977-017-0383-0

**Published:** 2018-01-08

**Authors:** Aneta Pluta, Lorraine M. Albritton, Marzena Rola-Łuszczak, Jacek Kuźmak

**Affiliations:** 1 OIE Reference Laboratory for EBL, Department of Biochemistry, National Veterinary Research Institute, Pulawy, Poland; 2 Department of Microbiology, Immunology and Biochemistry, College of Medicine, The University of Tennessee Health Science Center, Memphis, TN USA

**Keywords:** BLV, Deltaretrovirus, Env, Genetic variability, MEME, Conserved segments, Antigenic determinants, Positive selection

## Abstract

**Background:**

Bovine leukemia virus (BLV) is a deltaretrovirus infecting bovine B cells and causing enzootic bovine leucosis. The SU or surface subunit, gp51, of its envelope glycoprotein is involved in receptor recognition and virion attachment. It contains the major neutralizing and CD4+ and CD8+ T cell epitopes found in naturally infected animals. In this study, we aimed to determine global variation and conservation within gp51 in the context of developing an effective global BLV vaccine.

**Results:**

A total of 256 sequences extracted from the NCBI database and collected in different parts of the world, were studied to identify conserved segments along the *env* gene sequences that encode the gp51 protein. Using the MEME server and the conserved DNA Region module for analysis within DnaSP, we identified six conserved segments, referred to as A–F, and five semi-conserved segments, referred to as G–K. The amino acid conservation ranged from 98.8 to 99.8% in conserved segments A to F, while segments G to K had 89.6–95.2% conserved amino acid sequence. Selection analysis of individual segments revealed that residues of conserved segments had undergone purifying selection, whereas, particular residues in the semi-conserved segments are currently undergoing positive selection, specifically at amino acid positions 48 in segment K, 74 in segment G, 82 in segment I, 133 and 142 in segment J, and residue 291 in segment H. Each of the codons for these six residues contain the most highly variable nucleotides within their respective semi-conserved segments.

**Conclusions:**

The data described here show that the consensus amino acid sequence constitutes a strong candidate from which a global vaccine can be derived for use in countries where eradication by culling is not economically feasible. The most conserved segments overlap with amino acids in known immunodeterminants, specifically in epitopes D–D′, E-E′, CD8+ T-cell epitopes, neutralizing domain 1 and CD4+ T-cell epitopes. Two of the segments reported here represent unique segments that do not overlap with previously identified antigenic determinants. We propose that evidence of positive selection in some residues of the semi-conserved segments suggests that their variation is involved in viral strategy to escape immune surveillance of the host.

**Electronic supplementary material:**

The online version of this article (10.1186/s12977-017-0383-0) contains supplementary material, which is available to authorized users.

## Background

Bovine leukemia virus (BLV), which belongs to the *Deltaretrovirus* genus of the *Retroviridae* family, is the causative agent of Enzootic Bovine Leukosis (EBL) [1, 2]. The majority of BLV-infected cattle remain asymptomatic throughout their lives. However, about 5–10% of infected animals develop malignant tumors and 30% of infected individuals develop B-cell lymphocytosis characterized by extensive proliferation of CD5+ cells [3].

Similar to other retroviruses, the genome of BLV contains three major genes, *gag*, *pol* and *env*. The first two genes yield the virion structural proteins and the viral enzymes, including reverse transcriptase. The *env* gene encodes the envelope glycoprotein precursor (gp72), the viral membrane protein against which neutralizing antibodies would be expected to act. During synthesis, gp72 is cleaved into two associated components, a surface glycoprotein subunit of 51 kDa size (SU, gp51), implicated in receptor recognition and virion attachment, and a transmembrane glycoprotein subunit (TM, gp30) that induces the fusion of viral and cellular membranes necessary for virus to penetrate into host cell cytoplasm [4, 5].

The genetic variation in field isolates of BLV from naturally infected livestock represents a low mutation rate which appears to be less than that of other genera in *Retroviridae*. The genetic variability of BLV strains is typically analyzed by comparing the proviral sequences present in naturally infected cattle to the reference sequences of the FLK/BLV strain isolated from a BLV-infected foetal lamb kidney cell line (Accession no. M35242.1, USA) or to the field strain (Accession no. K02120, Japan). While the in vivo mutation rates measured on the *env* genes of BLV and lentiviruses are comparable at 0.009 and 0.0085 nucleotide changes per year, respectively, 81% of the nucleotide changes made by lentivirus reverse transcriptase (RT) are nonsynonomous whereas 50% of the BLV RT errors change the amino acid [6–9]. In vitro, the BLV RT is 10-fold more faithful than other retroviral polymerases and errs on average at an estimated one out of 20,800 nucleotides for an error rate of 4.8 × 10^−6^ nucleotides compared to 2.5–5.9 × 10^−4^ for purified HIV-1 RT and 3.4 × 10^−5^ measured during single cycle HIV-1 infection, 5.9 × 10^−5^ for avian myeloblastosis RT, 3.3 × 10^−5^ for Moloney murine leukemia virus RT, and 1.2 × 10^−5^ nucleotides for avian spleen necrosis virus RT [9–12].

Genetic studies have determined that the level of variation in proviral nucleotide and amino acid sequences is very low among infected cattle from geographically different parts of the world [13–27]. Phylogenetic analysis of the *env* gene of BLV isolates from these different geographical locations identified ten genotypes, G1–G10 [16]. Even among these ten genotypes the variation is low and only twenty amino acids among the 515 residues in gp72 differ among all the genotypes combined.

The lower non-synonomous mutation rate in BLV infection and very low interstrain variation in the envelope glycoprotein were predicted to be advantageous in developing a BLV vaccine [6]. A number of B-cell and T-cell determinants have been identified on gp51. The antigenic sites are the G, H, and F conformational epitopes, the A, B-B′, D–D′ and E-E′ linear epitopes and three neutralization domains ND1, ND2 and ND3 involved in virus neutralization and syncytium inhibition [28–31]. Five T-cell epitopes (CD4+ T-cell epitope, CD8+ T-cell epitope, gp51N5, gp51N11 and gp51N12) located within gp51 have been shown to be immunologic targets of cytotoxic T lymphocytes (CTL) and involved in induction of proliferative responses in BLV infected cattle [31–33].

The variation of these promising B-cell and T-cell determinants across global isolates has not yet been determined. Here we apply computational analysis of gp51 nucleotide and deduced amino acid sequences from the 256 BLV *env* gene sequences in the NCBI database to investigate global variation and conservation within SU. The results revealed the presence of highly conserved segments as well as several semi-conserved segments. Together these analyses define a hierarchy of conservation among the sequences of the B-cell and T-cell determinants that will be useful in developing an effective global BLV vaccine.

## Results

### Identification and selection of segments using MEME and DnaSP analyses

To analyze BLV isolates collected in different parts of the world we mapped conserved segments along the *env* gene sequence that encodes the gp51 protein. Two hundred and fifty-six sequences encoding residues 1–267 of the 268 amino acids in the mature gp51 surface subunit of BLV isolates representative for all known genotypes (G1–G10) were extracted from the NCBI nucleotide database and used for this study. Sequences were aligned and the alignment submitted to Multiple Expectation Maximization for Motif Elicitation (MEME) to identify sequence patterns or segments that occur repeatedly in the aligned group of 256 sequences analyzed. The MEME server generated twenty blocks of nucleotide sequence that occur once in the gp51 coding sequence and have E-values consistent with a very low probability that the MEME motif (term used interchangeably with a segment) is randomly present in the 256 BLV *env* genes. Eleven blocks were selected for further analysis based on considerations of four criteria: (1) low probability that the repeated occurrence of the sequence segment was random within the aligned coding sequences of gp51; (2) degree of amino acid conservation; (3) degree of nucleotide conservation; and (4) the genetic distance exhibited within a MEME motif. The eleven MEME motifs range in length from six to nineteen amino acids and have an average length just under twelve amino acids.

**Fig. 1:**
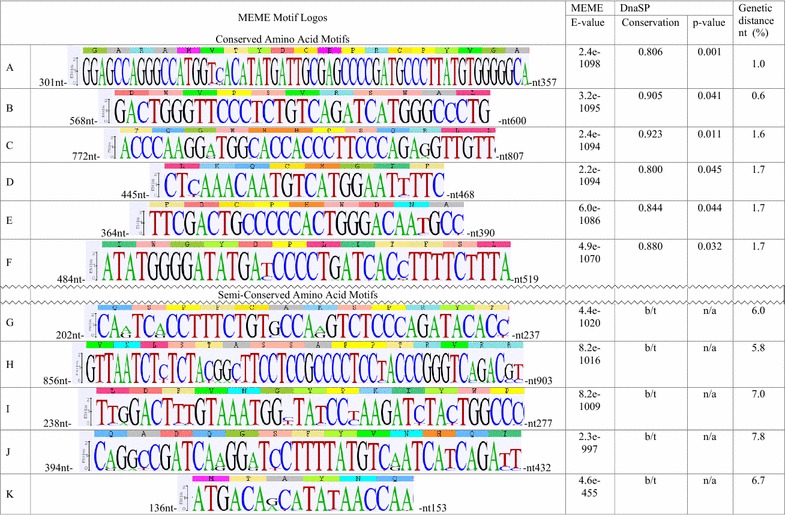
Sequence Logos of the predicted segments. WebLogo was used as described in Materials and Methods to generate a quantitative graphical representation of the 256 BLV nucleotide sequences in the multiple alignment from which MEME analysis identified conserved segments found in the gp51-encoding, 804 bp fragment of the *env* gene. The height of individual nucleotides within each Logo is proportional to the percentage of the 256 isolates in which that nucleotide occurred in that position. Nucleotides shown with maximum height were present at that position in all the isolates, whereas nucleotides with reduced height occurred in some but not all isolates. Nucleotides are numbered based on their position in the coding sequence of the envelope protein beginning with the “A” nucleotide of the start codon. Software parameters where chosen as followed: distribution of MEME motif occurrences (zero or one per sequence), number of different motifs (20), minimum motif width (15) and maximum motif width (60). DnaSP conservation values below the 80% or 0.800 threshold of conservation set for the analysis are indicated as b/t (below threshold) and their *p* values indicated as n/a (Not applicable). The genetic distance in each MEME block was calculated showing that MEME blocks A–F are conserved and MEME blocks G–K are highly variable.  %, resulting values of the mean genetic distances were multiplied by 100. Detailed data about nucleotides less frequently observed at specific positions of the selected segments are shown in Additional file 2: Table S2

Sequence Logos were generated to illustrate the consensus amino acid and nucleotide sequences of each MEME motif obtained using all BLV genotypes in the public databases (Fig. 1). The first six blocks identified by MEME analysis (referred to here as A–F) represent MEME motifs with E-values from 2.4e−1098 to 4.9e−1070. Blocks 16–20 (referred to here as G–K) showed MEME motifs with E-values from 4.4e−1020 to 4.6e−455. Blocks 7–15 were not further analyzed because they had intermediate characteristics. The amino acid conservation ranged from 98.8 to 99.8% in MEME motifs A to F. The remaining MEME motifs G to K had 89.6–95.2% conserved amino acid sequence.

The alignment was separately interrogated using the Conserved DNA Region module of DnaSP to identify segments of the nucleotide sequence conservation with the threshold for nucleotide conservation set at 80%. This analysis identified six statistically significant stable segments, with *p* value < 0.05 and nucleotide sequence conservation greater than or equal to 0.8 (80%). These segments were located exactly within the six blocks of segments A–F identified as nonrandom using MEME analysis. Segments G–J in MEME blocks 16–20 fell below the 80% nucleotide conservation threshold and their *p* values were not calculated (Fig. 1).

To estimate genetic distances for each of these MEME blocks *p* distance nucleotide substitution model in the Molecular Evolutionary Genetics Analysis software (MEGA) version 5.2.2. was used. The genetic distance relates to divergence between any two strands of aligned DNA sequences, and it is expressed as the number of nucleotide differences per site. In this study we calculated the overall mean, which is the arithmetic mean of all individual pairwise distances between the sequences under study. The six conserved segments had genetic distances from 0.6 to 1.7%, while distance values from 5.8 to 7.8% characterized segments G–K (Fig. 1). Based on these findings taken together, we grouped segments A–F together as conserved in amino acid and nucleotide sequence and segments G–K as semi-conserved in amino acid sequence.

### Evidence of purifying selection in segments A–F and positive selection in segments G–J

Genetic diversity within a coding sequence from a population of related sequences can be assessed by comparing the rates of synonymous and non-synonymous substitutions per site. An excess of non-synonymous mutations suggests selection for change, or positive selection, which is often the result of an antagonistic interaction between a virus and its host. This high genetic variation confers a fitness advantage to the pathogen in its attempt to evade host defenses. In contrast, an excess of synonymous mutations suggests that there is selection against change, or purifying selection, meaning the sequence is likely to be beneficial to the host. The negatively selected sites point to functional constraint, and could be used in vaccine design. The high degree of amino acid and nucleotide sequence conservation of segments A–F among the 256 BLV sequences suggested that these portions of the *env* gene are very stable during replication and might be undergoing negative or purifying selection associated with predominantly synonymous substitutions. Whereas, the nucleotide variation of segments G–J suggested the possibility that they contained sequences that are less stable and under positive selection. To examine this possibility, the frequency of non-synonymous (dN) and synonymous (dS) nucleotide substitutions per site within each segment was calculated and the ratio dN/dS was estimated.

Selection analysis revealed that the rates of synonymous nucleotide substitutions per site (dS) were higher than the non-synonymous rates (dN) within all analyzed segments showing a high probability of purifying selection. These differences were statistically significant as judged by Z-test (Z-test: *p* < 0.0001 for A-J segments and *p* < 0.05 for K segment). The dN/dS values were substantially less than 1 for all segments, also in agreement with the existence of strong purifying selection pressure. Nevertheless, dN/dS values noted for semi-conserved segments G–K were substantially higher than those noted for conserved segments A–F, suggesting the existence of putative sites undergoing positive but not purifying selection.

**Fig. 2:**
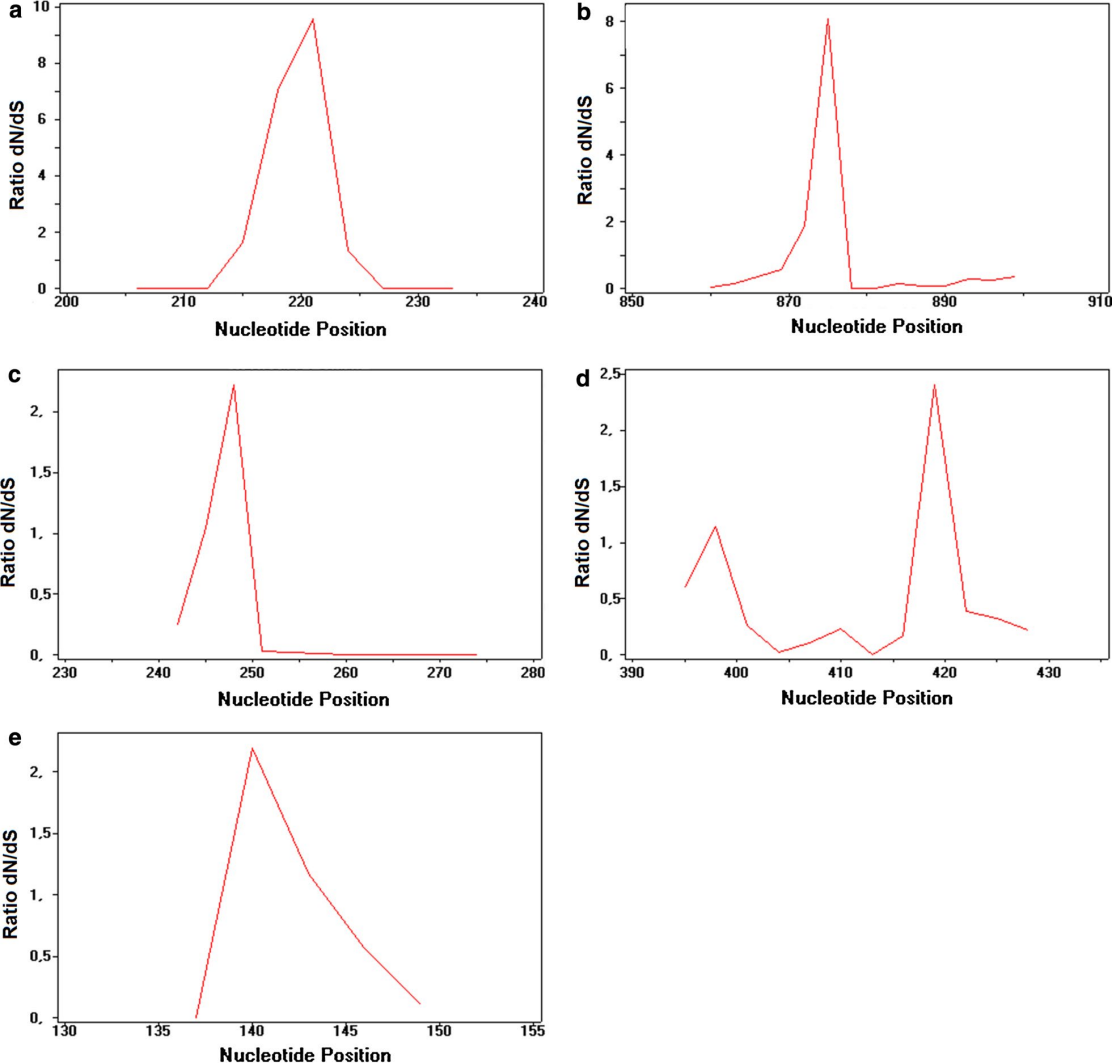
Sliding window analysis based on the rate of DNA polymorphism and divergence in synonymous sites and non-synonymous sites. Values shown on the y-axis are the ratio of the rate of non-synonymous substitutions dN to the rate of synonymous substitutions dS at each nucleotide position calculated using the following sliding window options: window length: 9, step size: 3. The segments analyzed were: **a** 202–237 (segment G), **b** 856–903 (segment H), **c** 238–277 (segment I), **d** 394–432 (segment J), **e** 136–153 (segment K). Nucleotide positions were numbered beginning with the adenosine residue in the first position of the ATG start codon of the gp51 coding sequence

To test this possibility, sliding window analysis was performed and the rate of DNA polymorphism and divergence per codon was calculated. A standard indication of selective pressure is the ratio of dN/dS, where dN/dS ~ 1 signifies neutral evolution or a balance in evolutionary pressure on that sequence, dN/dS < 1 indicates negative or purifying selection and a ratio > 1 indicates positive selection pressure [5]. The following segments with putative positive selection sites within particular semi-conserved segments were identified: 213–228 nt, 861–879 nt, 238–252 nt, 394–402 nt, 414–432 nt and 138–150 nt for segments: G–K, respectively, based on the ratio of synonymous sites (dS) to nonsynonymous sites (dN) within each codon (Fig. 2). While the majority of codons located in these segments had elevated dN/dS ratios that were nonetheless < 1, six codons in segments G–K had dN/dS ratios > 1 identifying them as the major sites where positive selection occurs. These were codons 74 in segment G, 291 in segment H, 82 in segment I, 133 and 141 in segment J and 48 in segment K (Table 1).

**Table 1:** Computational analyses indicate that conserved MEME motifs G–K of gp51 are under positive selection

MEME motift^a^	dS^b^	dN^c^	Z-test	dN/dS ratio	Sites of positive selection^d^	*p* values^e^
A	0.03450	0.00629	< 0.0001	0.182	0	NA
B	0.03541	0.00253	< 0.0001	0.071	0	NA
C	0.08121	0.01129	< 0.0001	0.139	0	NA
D	0.09827	0.00000	< 0.0001	0.000	0	NA
E	0.09267	0.00133	< 0.0001	0.014	0	NA
F	0.09420	0.00000	< 0.0001	0.000	0	NA
G	0.11690	0.02867	< 0.0001	0.245	Lys^74^, Arg^74^	< 0.05
H	0.09437	0.04350	< 0.0001	0.461	Ala^291^, Val^291^, Thr^291^	< 0.05
I	0.24183	0.01934	< 0.0001	0.080	Phe^82^, Ser^82^, Leu^82^	< 0.05
J	0.14623	0.05945	< 0.0001	0.381	Val^133^, Thr^133^, Pro^133^, Asp^133^, Ala^133^, Asp^141^, Asn^141^	< 0.05
K	0.10056	0.05619	< 0.050	0.559	Thr^48^, Tyr^48^, Val^48^, Ile^48^	< 0.05

^a^MEME motifs A–F are highly conserved at the amino and nucleotide sequence levels, while MEME motifs G–K are conserved at the amino acid but not the nucleotide level

^b^dS, rate of synonymous substitutions

^c^dN, rate of non-synonymous substitutions

^d^Positions of positive selection sites are the amino acid numbers beginning from the N-terminus of the precursor

^e^*p*-values were calculated for the positive selection sites; NA, not applicable

### Refinement of the gp51 sequence segment map

In the process of evaluating the sequence segments (MEME motifs), we placed them on the consensus amino acid sequence generated in the initial MEME analysis. It was then apparent that two pairs of conserved MEME motifs, e.g., A and E, and D and F, and one pair of semi-conserved MEME motifs, e.g., G and I, were located very close to each other in the primary sequence. Any of these pairs might actually represent a larger segment that was split up because the maximum MEME motif width was set at 60 nucleotides or 20 amino acid codons in our initial MEME analysis. To determine if this was the case, the 256 gp51-encoding sequences were submitted a second time to the MEME server using a minimum MEME motif width of 60 nucleotides and a maximum width of 99 nucleotides (20–33 amino acid codons). Using these parameters, the output should cover the 268 codons of gp51 almost twice over (minimum length of 20 codons × 25 motif block output = 500 codons). Since all of the segments discovered in the initial analyses are shorter than the minimum 20 amino acid width in the new analysis, we expected that the original A-K MEME motifs would be included in the blocks from this new analysis only if they are part of longer conserved regions.

Six blocks were identified by MEME using the wider motif parameter and named MEME motifs I–VI (Table 2). E-values ranged from 6.7e−2042 to 7.5e−1028 and amino acid conservation ranged from 94.4 to 99.6%. Genetic distances ranged from 1.2 to 4.6%, however, only MEME motifs I, IV and V had *p* values < 0.05 for nucleotide conservation indicating that these were statistically significant stable nucleotide segments. Eight short MEME motifs (A–G and I) are part of five of the longer MEME motifs (I–III and V–VI). Three of the longer MEME motifs contain adjacent shorter MEME motifs found in the initial analysis. Among the initial eleven MEME motifs discovered, H, J and K were not found to be part of longer MEME motifs. Long segments discovered in the second MEME and DnaSP analyses of the BLV gp51 *env* sequences are highly conserved at the amino acid level but less well conserved at the nucleotide level (Table 2).

**Table 2:** Long conserved MEME motifs found in BLV gp51 *env* sequences

Block	MEME Motif	MEME E-value	Amino acid conservation (%)	Genetic distance (%)	Nucleotide conservation	*p* value
1.	I	6.7e−2042	98.0	1.2	0.813	0.007
2.	II	8.4e−2032	99.5	2.3	0.800	0.063
3.	III	9.7e−2032	99.1	3	0.800	0.056
4.	IV	1.8e−2099	99.6	1.3	0.807	0.037
5.	V	5.4e−2042	97.3	2.5	0.818	0.029
6.	VI	7.5e−1028	94.4	4.6	b/t	n/a

b/t, value is below threshold of 80% conservation or 0.80

n/a, not applicable

%, resulting values of mean genetic distances were multiplied by 100

### Relationship of segments to known and predicted functional domains of gp51

**Fig. 3:**
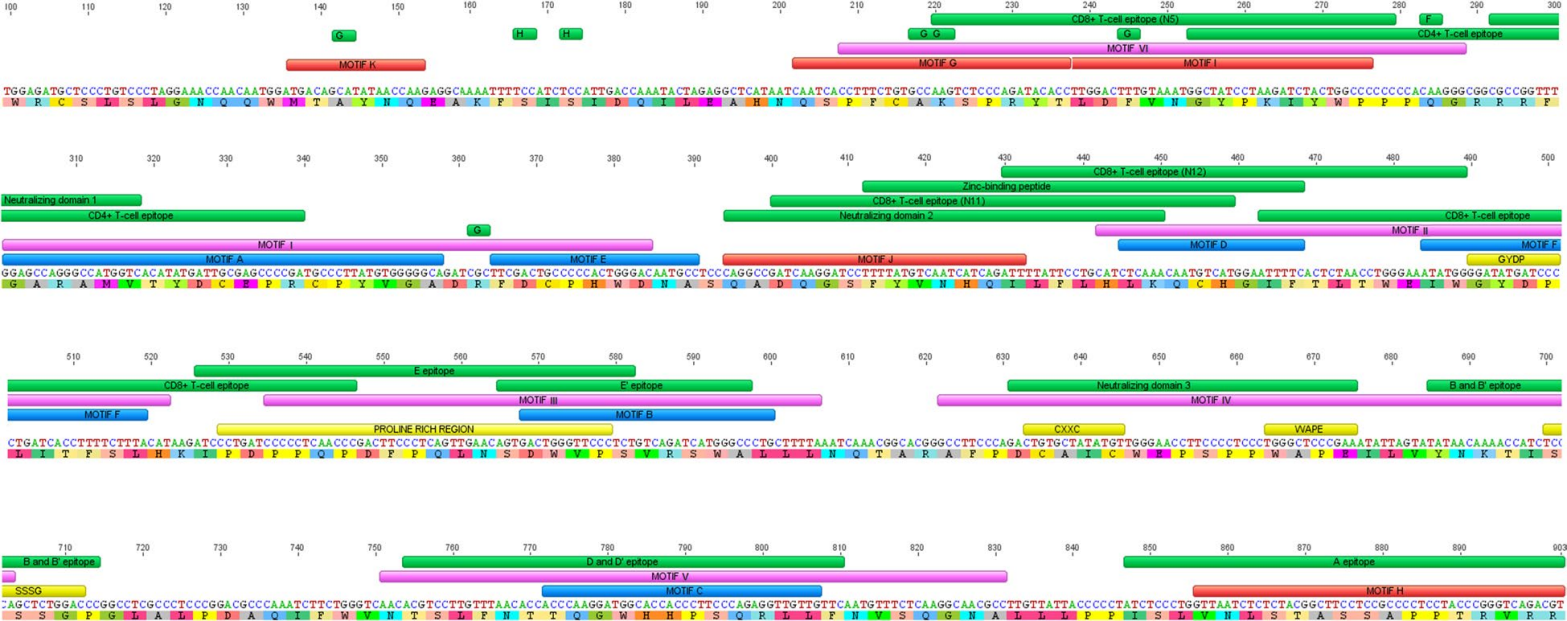
Distribution of segments along the surface glycoprotein gp51. The labeled green bars in the upper part of figure refer to the coding sequences of antigenic determinants and to the Zinc-binding peptide. MEME motifs obtained by the initial MEME analysis with a motif width range set at 5–20 amino acids are indicated by blue (A–F) and red (G–K) bars above the consensus sequence. MEME motifs I–VI defined in the second MEME analysis using a motif width range of 20–33 amino acids are indicated by purple bars. Epitopes B, B′, E, and E′ (linear), G, H and F (conformational), ND1, 2, and 3 (neutralization domains), and B-cell and T-cell determinants are indicated by green bars. Previously reported conserved functional sequence segments and the putative proline rich region are indicated by yellow bars above the sequence. The sequence of mature gp51 is shown. Nucleotides are numbered based on their position in the coding sequence of the envelope protein beginning with the “A” nucleotide of the start codon

To determine possible association of segments with functional domains within gp51 glycoprotein, the segment sequences were aligned with the nucleotide and amino acid MEME consensus sequence (Fig. 3). Short segment K is located near the N-terminus of gp51. While this segment encompasses a single residue of conformational epitope G, represented by Tyr-48, it is otherwise unique in its lack of association with known antigenic determinants and function. The other segments overlap with previously identified functional and immune recognition sequences. Conserved segments A–F are located within the central and the C-terminal part of gp51 protein between amino acids 105–280, overlapping mainly linear and T-cell epitopes. In addition, segment A overlaps with the C-terminal portion of the B cell epitope (gp51p16-19) described by Bai et al. in 2015 [33].

While the three-dimensional structure for a portion of TM proteins from BLV (gp30) and the human deltaretrovirus HTLV-1 (gp21) have been solved, no structure is available for gp51 or the SU (gp46) from HTLV-1. However, the envelope glycoproteins of both retroviruses belong to the cd09948:Ebola_RSV-like_HR1-HR2 and pfam00429:TLV_coat ENV polyprotein (coat polyprotein) families of glycoproteins which include the envelope glycoproteins of HTLV-1, BLV, the avian retroviruses Rous sarcoma virus and Avian Leukosis Virus subgroup J, Friend Murine Leukemia Virus (Fr57 MLV) and Feline leukemia virus type B (FeLV-B) and Ebola virus [34, 35]. Members of these families contain related functional domains of similar sequence and there is evidence that they use a shared mechanism for attachment and membrane fusion during virus entry based on isomerization of a disulphide bond between the attachment and fusion subunits [35]. Structures from three members of the families have been solved, e.g., that of the established receptor binding domains of gammaretroviruses Fr57 MLV and FeLV-B, and of the ectodomain of the mature envelope glycoprotein from Ebola virus.

**Fig. 4:**
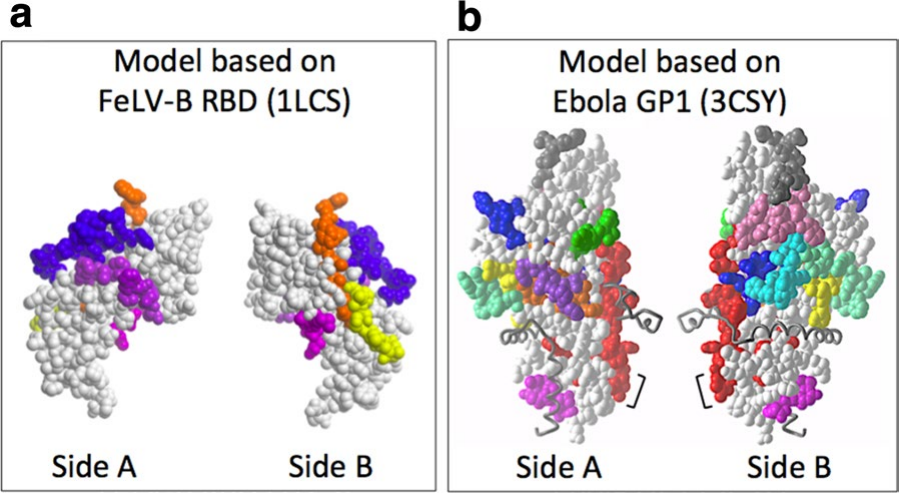
Representation of the conserved and semi-conserved segments on 3 D glycoprotein structure models of gp51. Homology models are shown as space-filled images of opposite sides arbitrarily named side A and side B. Each is based on alignment of residues 1–156 of mature gp51 with the analogous sequences of FeLV-B RBD (accession number 1LCS), or alignment of residues 1–298 with the amino acid sequence of Ebola Zaire GP1 (accession number 3CSY). The model of the gp51 RBD contain conserved segments A (blue), E (violet), D (yellow), and F (orange), and semi-conserved segments K (magenta), G (cyan), I (light green), and J (light blue). The model of the entire gp51 based on Ebola GP1 contains these segments plus segments included in residues 156–298, specifically conserved segments B (green), IV (red), and C (pink), and semi-conserved segment H (gray)

Using homology modeling based on the gammaretroviral receptor binding domain (RBD) structures, we placed the conserved segments within residues 1- 156 of mature gp51 in three-dimensional structural models. A previously published BLV model based on Fr57 MLV structure was supported by natural variants in BLV envelope protein, but was recognized as flawed because there were several small gaps in the main peptide chain of the BLV RBD that the model could not resolve [34]. The subsequent publication of the FeLV-B RBD structure (1LCS) provided a second template structure upon which the BLV RBD could be modeled. Figure 4a shows a model based on the FeLV-B structure which is reported here for the first time. This new model resolves the gaps in the BLV RBD peptide chain that were the weakness of the previous model. The predicted locations of surface-exposed residues in the conserved and semi-conserved segments identified in the analyses reported here are shown in colors by motif segment.

While highly speculative, a model based on the alignment of conserved domains previously identified in the cd09948:Ebola_RSV-like_HR1-HR2 and pfam00429:TLV_coat ENV polyprotein (coat polyprotein) families [35] was also generated. Since the Ebola glycoprotein GP structure is the only structure that includes more than the RBD for a member of these families, its use as the model template afforded an opportunity to model the six segments which are carboxy-terminal to residue 156 of mature gp51 and lie outside the alignments with the RBD of FeLV-B and Fr57 MLV (Fig. 4b). It also could provide insight into segments that are surface-exposed in the RBD model but which are buried in gp51 by residues 197–267 of gp51. This model predicts that the majority of residues within segments A–F are exposed on the surface of the gp51 glycoprotein, including the following residues: 106-VTYDCEPRCPY-116 of segment A, 194-PSVRS-197 of segment B, 256-TQGWHHPSQRLL-269 of segment C, as well 164-GYDP-167 of segment F. Conversely, conserved segments D and E as well as residues 198-WALLLNQ-204 in segment B are predicted to be buried in a beta-strand within the core of the structure in the model shown in Fig. 4b.

## Discussion

Since the discovery of BLV as the causative agent in EBL in 1969 [36], efforts at global elimination of BLV have centered on testing and removal of infected animals from herds as the preferred method of eradication of the disease. This strategy has achieved eradication in some countries particularly those with a low initial prevalence of BLV-infected cattle. However, EBL remains endemic in locations where the high prevalence rate is an economic barrier to the culling strategy and large numbers of cattle exhibit the most common clinical manifestation of BLV infection, persistent lymphocytosis. These locations include North and South America, Middle Eastern and Southeast Asian nations and the Caribbean region (OIE World Animal Health Organization from the years 2015 and 2016). The genetic selection of cattle resistant to BLV infection might be beneficial for minimizing economic losses by reducing disease severity but not for eradication because it currently appears that complete resistance to BLV cannot be obtained by selection of cattle with BLV-resistant alleles of the DRB3.2 gene [37, 38].

A vaccine that protects against BLV transmission would provide an economical alternative method of eradication in countries with high prevalence rates, but the prospect for a preventive or therapeutic anti-deltaretrovirus vaccine are uncertain [39, 40]. One way to make an anti-BLV vaccine would be a peptide-based strategy consisting of highly conserved immunogenic peptide constructs capable of eliciting broadly neutralizing antibody responses [40–42].

The initial goals of our study were to determine a consensus gp51 amino acid sequence for BLV isolates from endemic locations all over the world, identify which, if any, of the known BLV antigenic determinants within glycoprotein gp51 are conserved globally within and between all known genotypes, and to gain insights into the evolution of BLV gp51 during natural in vivo infection particularly with respect to known neutralizing domains and antibody and T-cell epitopes. Toward this objective, all primary isolate sequences that contain at least the coding sequence of residue 1–297 of the 298 residues in mature gp51 were mined from the NCBI public database and submitted to computational analysis from which a consensus amino acid sequence was obtained (Additional file 1: Table S1).

Based on the results reported here, we draw five conclusions. First, we conclude that the consensus amino acid sequence derived from MEME analysis represents the primary consensus sequence of global BLV gp51. The set of 256 proviruses used to generate the consensus sequence represents nucleotide sequences isolated from infected cattle in twenty-one countries and includes isolates from all of the ten known BLV genotypes.

Second, we propose to classify the new segments exhibiting DNA conservation greater than or equal to 80% and genetic distances ranging less than or equal to 3.0% as conserved, and those showing less than 80% DNA conservation and a somewhat higher degree of variability greater than or equal to 4.6% as semi-conserved. Based on these criteria, gp 51 contains six highly conserved segments and five semi-conserved segments of five to twenty residues in length that are distributed throughout gp51. Two pairs of conserved segments (A and E, and D and F) were found to be part of larger conserved segments I and II, respectively, when the analysis parameters were set for segments of longer length (20–33 amino acids) than in our initial analyses. Similarly, semi-conserved segments G and I were found to be part of a larger semi-conserved segment VI.

Third, we conclude that among the segments reported here, only the conserved ones exhibited evidence of purifying selection of their sequences, e.g., dN/dS ratios of substantially less than 1 (≤ 0.182). These relatively low levels of variability reflect a general feature of BLV genomes, that is, its characteristically high degree of conservation among different strains. Low variability among strains is also characteristic of other viruses belonging to the genus *Deltaretrovirus* [6, 12]. It was recently shown that the homology of the *env* gene was 94.5–97.7% amongst BLV isolates representative of the ten genotypes [16]. This level of variability is much lower than that reported for conserved regions identified in studies by others of human cytomegalovirus or herpes simplex virus type 1 [43, 44].

We favor that the purifying selection during evolution of the conserved segments suggests that these segments may contain critical core structure sequences, or sequences that interface with the TM gp30, or codons for the residues that interact with the BLV entry receptor. The homology models predict that part of the core structure of the RBD is formed by conserved segments D and F. In the models these segments form adjacent beta-strands previously proposed to face inward toward the trimer interface [35]. The presence of the previously recognized small motif CXXC in large conserved segment IV identifies this segment as containing residues in the gp51:gp30 heterodimer interface. In native envelope glycoprotein on virions, one of the cysteine residues in CXXC motif of gp51 is covalently bound to a cysteine in the conserved CX_6_CC motif of gp30 and makes an essential contribution to the association of gp51:gp30 heterodimers [45, 46]. In addition, the C-terminal domain of HTLV-1 surface subunit has been reported to be involved in transducing the envelope activation signal upon receptor binding [47, 48], suggesting that conserved segment C in the analogous domain of gp51 may also participate in activation of membrane fusion.

With respect to residues that interact with the BLV entry receptor, neither the receptor nor the gp51 residues with which it interacts are known. However the location of the BLV RBD within the amino-terminal 156 residues of the mature BLV gp51 subunit is supported by homology to the established RBD of the HTLV-1 and the gammaretroviruses [35, 49, 50] and the positions of the proline rich region and the conserved CXXC motif in gp51 [46]. Assuming that all strains of BLV use a common entry receptor, it is most likely that the receptor binding site lies within a highly conserved segment. Four conserved (A, D, E and F) and four semi-conserved segments (G, I, K and J) lie within the first 156 residues (Fig. 3).

We favor that the binding site for the receptor includes conserved segments A and E for the following reasons. First, the receptor binding site is likely to be within or adjacent to a neutralizing domain. There are two neutralizing domains within residues 1–156, ND1 and ND2. Two segments overlap with these domains: segment A overlaps with the C-terminal two-thirds of ND1 and segment J overlaps with the N-terminal two-thirds of ND2. Second, the receptor binding site is likely to be located on the analogous surface to that of the established sites on the Fr57 MLV and Fe-LV-B RBDs. The homology models of gp51 based on these two RBD structures predict that segment A lies on the analogous surface whereas all but the first three residues of segment J are predicted to be buried within the core of the model. Lastly, while segment E is not included in a known neutralizing domain, the homology models predict that it lies beside segment A and that together they form a convex surface comparable in size and predicted location to the known receptor binding sites of Fr57 MLV and FeLV-B. In agreement with this last prediction by the models, analysis for segments of 20–33 amino acid length identified segments A and E as belonging to a single long conserved segment I.

Fourth, we conclude that amino acid positions 48 in segment K, 74 in segment G, 82 in segment I, 133 and 142 in segment J, and residue 291 in segment H are undergoing positive selection. Each of the codons for these six particular residues contains the most highly variable nucleotides within their respective semi-conserved segments. We favor that the genetic evidence of positive selection in these residues is consistent with viral evolution to escape immune surveillance as judged by the location of each of the six residues within known epitopes and neutralizing domains. Residues 48, 74 and 82 comprise three of the four amino acids in conformational epitope G, residues 133 and 142 are located in ND2 (which overlaps with the Zinc binding peptide), and residue 291 is in epitope A.

It has been postulated that some divergent residues within conserved segments may be under positive selection [32, 51]. Zhao and coworkers investigated possible selection over the entire glycoprotein sequence from forty-four unique isolates and found evidence of negative selection in the coding sequences for most of the known functional domains. However, those authors found evidence of positive selection only in the immunostimulatory conformational epitope G and linear epitopes D, D′ and concluded that these segments and not others were under selection for immune escape. Using a different approach to study selection in natural BLV infection, that is, analysis of individual conserved and semi-conserved segments instead of analysis of the whole glycoprotein, we confirmed that residues in conformation epitope G are under positive selection but found no evidence of positive selection in segment C which comprises a large portion of linear epitopes D, D′. In contrast to their finding of no evidence for positive selection of residues within other segments of gp51, we found evidence that ND2 (which includes segment J) and epitope A at the C-terminal end of gp51 (which includes segment H) are undergoing positive selection. We believe that the difference between our reports attributes to the segment-based analysis reported here being a more sensitive method for identifying rare residues of BLV that are under selection but in otherwise highly conserved segments.

Lastly, we conclude that the consensus amino acid sequence constitutes a strong candidate from which a universal vaccine can be derived for use in countries where eradication by culling is not economically feasible. Two of the MEME motifs represent unique segments that are not contained within previously identified immune determinants. Based on their location relative to that of known functional and structural domains in other retroviral SU, we propose that one (segment K) of these newly identified segments is involved in the folding of native gp51 and the other (segment E) is a strong candidate for part of the receptor binding sequence. Notably, none of the conserved or semi-conserved segments include residues in conformational epitope H. Taken together the results suggest that globally dispersed variations in segments E and K and in epitope H sequences make them poor candidates for use in a peptide vaccine strategy. In contrast, highly conserved segment A, which is another strong candidate for the receptor binding site, contains previously identified neutralizing and T- cell determinants that suggest it is a good candidate for use in a peptide vaccine, one capable perhaps of eliciting neutralizing antibody responses.

All of the B-cell and T-cell determinants and neutralizing domains, and all but one (H) of the antigenic epitopes contain and overlap with the new segments reported here. The new semi-conserved segments overlap with conformation epitopes G and F, linear epitopes A and B-B′, neutralizing domains 2 and 3, CD4+ T-cell epitopes N5 and N11. Of the three amino acid differences between the consensus sequence and that of strain FLK, two residues (74 and 82) are in the CD8+ T-cell epitope N5 and in conformational epitope G and the third is in the D, D′ epitope. This low level of difference is in line with the unusually high fidelity of the BLV polymerase and high level of conservation seen across global isolates. The most conserved segments encompass amino acids in epitopes D–D′, E-E′, two CD8+ T-cell epitopes, neutralizing domain 1 and two CD4+ T-cell epitopes, suggesting that their consensus sequence has limited global variation and are prime candidates for development of an globally effective peptide vaccine.

## Methods

### Sequence motif discovery

MEME version 4.10.0 [52], a hidden Markov model based software, and DnaSP version 5.10 software [53] were used to identify segments that occur in a group of 256 sequences extracted from the NCBI nucleotide database and represent all known BLV *env* sequences. Seventy-one proviruses were from three East Asian countries (Myanmar, South Korea and Thailand), sixty-three were from six South American countries (Argentina, Boliva, Brazil, Peru, Paraguay and Uruguay), thirty-nine from Russia, thirty-four from Japan, twenty-six from four European countries (Belgium, Italy, Moldova and Poland), eighteen from three North American sites (Caribbean, Costa Rico and the United States), and five proviruses from Australia, Iran and the Ukraine combined (Additional file 1: Table S1). Sequences derived from PCR-amplification of proviruses using the nested primer sets related to those introduced by Fechner et al. in 1997 were excluded from the analysis because they comprise fragments of < 500 bp and encode only the central half of gp51 [54]. The coding sequence for the 33 amino acid signal peptide was also excluded from analysis because the majority of the sequences in the database that encode the entire mature gp51 are derived from an 804 bp restriction fragment that does not contain signal peptide coding sequence.

The following steps were performed during the MEME and DnaSP analysis. First, a multiple sequence alignment was generated using ClustalW implemented in Geneious Pro 5.5.9 Software (Biomatters Ltd). Second, the aligned sequences were submitted to the MEME server (http://meme-suite.org/tools/meme). The MEME analysis was run in zoops (zero or one per sequence) mode with the maximum number of motifs set at 20. The optimal motif width range was set between 15 and 60 nucleotides (*i.e.*, 5 and 20 amino acids in length) to reflect the length of the synthetic peptides commonly used in epitope mapping as well as the typical number of amino acid (aa) residues identified in antigenic determinants. The E-values are the estimated log likelihood ratio of a sequence pattern calculated by MEME analysis as a measure of how different each nucleotide in the segment (motif) is from an n-order Markov model matrix of the same width but randomized sequence. A segment with an E-value below e^−9.2^ has an estimated < 0.0001 probability that its sequence occurs randomly within the aligned input sequences. Third, the alignment was separately imported into the Conserved DNA Region module implemented in DnaSP Ver. 5.10.01 software and the minimum conservation threshold set at 80%. Lastly, the combined analyses were used to identify conserved segments of interest in the *env* genes.

Weblogo was then used to generate a quantitative graphical representation of the segments and sequence alignments. To estimate the level of nucleotide sequence divergence for conserved and variable segments, maximum composite likelihood model analysis (MEGA 5.2.2) was conducted as described [55]. Synonymous and non-synonymous sites were identified using the DnaSP 5.0. Statistical analyses identifying synonymous and non-synonymous sites was performed using STATISTICA ver. 10 (StatSoft, Dell Software, USA). Specifically, the number of synonymous substitutions per synonymous site (dS), the number of non-synonymous substitutions per non-synonymous site (dN) and their variances were calculated and applied to a selection Z-test to evaluate whether the segments were under positive or purifying selection. For comparison of two paired, nonparametric groups of data points, dN versus dS, a Wilcoxon matched pairs test was used to calculate the *p* values. Based on the results of the first analyses, the 256 member sequence alignment was submitted to the MEME server again using zoops mode with the maximum number of motifs set at 25 and the optimal motif width range set between 60 and 99 nucleotides (i.e., 20 and 33 amino acids in length) and the motif block output was subjected to further analysis as described above.

### Molecular modeling

The consensus amino acid sequence for residues 3–156 comprising the putative RBD of mature SU sequence derived in the MEME analysis was aligned with the analogous extracellular amino acid sequence of the two gammaretroviral SU whose crystal structure has been solved, e.g., residues 9–236 of the mature SU from Fr57 MLV isolate 57 [56] and residues 4–207 of the mature SU from FeLV-B virus [57]. Each alignment was submitted for molecular modeling [58] using the 2.0 Å structure of the Fr57 MLV RBD (1AOL) and the 2.5 Å structure of FeLV-B RBD (1LCS) as templates. Noting that the BLV envelope glycoprotein is a member of the HTLV-1 superfamily of envelope glycoproteins [35], residues downstream of BLV gp51 residue 156 were modeled based on the known structure of the only member of the HTLV-1 superfamily for which structure of the analogous residues downstream of the RBD is available, e.g., GP of the Zaire strain of Ebola virus. Alignments of the consensus BLV gp51 sequence and that of Ebola virus GP were submitted for molecular modeling [58] using the 3.4 Å structure of the pre-fusion GP1-GP2 trimer (3CSY) [59] and the 3.3 Å structure of the host-primed GP1 and GP2 complexed with the virus binding domain of its host cell fusion receptor Niemann-Pick C1 (5HJ3) [60]. Visualization of the predicted structures and figure preparation were performed using the MolSoft ICM Browser. Conserved and semi-conserved segments were visualized on structure models of the envelope protein.

## Additional files

**Additional file 1: Table S1.** Accession numbers and country or region of origin of the sequences analyzed in the study. §—direct submission to GenBank.

**Additional file 2: Table S2.** The conserved and semi-conserved segments in BLV envelope glycoprotein. A white color reflect segments highly conserved at the amino and nucleotide sequence levels, dark yellow color reflect segments conserved at the amino acid level. # The sequences are from MEME output and they reflect the nucleotides that are most frequent at these positions. For example, having a T in the output sequence does not mean that all gp51 *env* segments of BLVs will have a T nucleotide at that position. A [|] indicates more than one nucleotide is frequent at this position. A “-“ horizontal dashes between nucleotides denote the next codon. *The amino acid residues that are most frequent at these positions.
