# Supplementary material for: Computational analysis of envelope glycoproteins from diverse geographical isolates of bovine leukemia virus identifies highly conserved peptide motifs
Source: Retrovirology. 2018 Jan 8;15:2. doi: 10.1186/s12977-017-0383-0 (PMC5759284; doi:10.1186/s12977-017-0383-0)
Supplement: Supplementary file 1 — Additional file 1: Table S1. Accession numbers and country or region of origin of the sequences analyzed in the study. §—direct submission to GenBank. [file 12977_2017_383_MOESM1_ESM.docx]

**Supplementary Table 1. Accession numbers and country or region of origin of the sequences analyzed in the study.**

| No | Acc. Number | Origin | Genotype | References |
| --- | --- | --- | --- | --- |
| 1 | K02120.1 | Japan | 1 | Sagata, 1985 [1] |
| 2 | AF257515.1 | Argentina | 2 | Dube, 2000 [2] |
| 3 | HE967301.1 | Uruguay | 1 | Moratorio, 2013 [3] |
| 4 | HE967302.1 | Uruguay | 1 | Moratorio, 2013 [3] |
| 5 | HE967303.1 | Uruguay | 1 | Moratorio, 2013 [3] |
| 6 | M35238.1 | Belgium | 4 | Mamoun, 1990 [4] |
| 7 | M35240.1 | Belgium | 4 | Mamoun, 1990 [4] |
| 8 | M35239.1 | USA | 1 | Mamoun, 1990 [4] |
| 9 | K02251.1 | Belgium | 4 | Rice, 1984 [5] |
| 10 | AF033818.1 | USA | 4 | Petropoulos, 1997 [6] |
| 11 | AB934282.1 | Japan | 1 | Mekata, 2015 [7] |
| 12 | LC005616.1 | Japan | 1 | Polat, 2017 [8] |
| 13 | LC005615.1 | Japan | 1 | Polat, 2017 [8] |
| 14 | LC164084.1 | Japan | 3 | Murakami, 2016 [9] |
| 15 | LC164085.1 | Japan | 1 | Murakami, 2016 [9] |
| 16 | LC164086.1 | Japan | 1 | Murakami, 2016 [9] |
| 17 | AF067081.1 | Poland | 4 | Limansky, 2002 [10] |
| 18 | AY995174.1 | South Korea | 1 | Lee, 2015 [11] |
| 19 | LC154066.1 | Myanmar | 10 | Polat, 2017 [8] |
| 20 | LC154064.1 | Myanmar | 10 | Polat, 2017 [8] |
| 21 | LC154067.1 | Myanmar | 10 | Polat, 2017 [8] |
| 22 | LC154065.1 | Myanmar | 10 | Polat, 2017 [8] |
| 23 | D00647.1 | Australia | 1 | Coulston, 1990 [12] |
| 24 | JF720354.2 | Russia | 4 | Pluta, 2012 § |
| 25 | KF801467.2 | Moldova | 7 | Pluta, 2017 [13] |
| 26 | JF720350.2 | Russia | 7 | Pluta, 2012 § |
| 27 | HM563758.3 | Ukraine | 7 | Rola-Łuszczak, 2013 [14] |
| 28 | KF801468.2 | Moldova | 7 | Pluta, 2017 [13] |
| 29 | JF720351.2 | Russia | 7 | Pluta, 2012 § |
| 30 | HM563764.3 | Ukraine | 8 | Rola-Łuszczak, 2013 [14] |
| 31 | JF720352.2 | Russia | 7 | Pluta, 2012 § |
| 32 | JN695882.1 | Russia | 7 | Lomakina, 2014§ |
| 33 | HM563779.3 | Russia | 4 | Pluta, 2011§ |
| 34 | HM563749.3 | Russia | 7 | Rola-Łuszczak, 2013 [14] |
| 35 | JF720353.2 | Russia | 7 | Pluta, 2011§ |
| 36 | HM563754.3 | Russia | 7 | Pluta, 2011§ |
| 37 | KF801469.2 | Moldova | 7 | Pluta, 2017 [13] |
| 38 | HM563783.3 | Russia | 4 | Pluta, 2011§ |
| 39 | JF720355.2 | Russia | 4 | Rola-Łuszczak, 2012§ |
| 40 | KF801470.2 | Moldova | 7 | Pluta, 2017 [13] |
| 41 | JN695880.1 | Russia | 7 | Polat, 2017 [8] |
| 42 | JF720356.2 | Russia | 4 | Rola-Łuszczak, 2012§ |
| 43 | KF801459.2 | Moldova | 4 | Pluta, 2017 [13] |
| 44 | JN695879.1 | Russia | 7 | Lomakina, 2014§ |
| 45 | KF801460.2 | Moldova | 4 | Pluta, 2017 [13] |
| 46 | KF801457.1 | Moldova | 7 | Pluta, 2017 [13] |
| 47 | KF801464.2 | Moldova | 7 | Pluta, 2017 [13] |
| 48 | KF801461.2 | Moldova | 7 | Pluta, 2017 [13] |
| 49 | JN695881.1 | Russia | 7 | Lomakina, 2014§ |
| 50 | KF801458.1 | Moldova | 7 | Pluta, 2017 [13] |
| 51 | KF801465.2 | Moldova | 7 | Pluta, 2017 [13] |
| 52 | KF801466.2 | Moldova | 7 | Pluta, 2017 [13] |
| 53 | JN695878.1 | Russia | 4 | Lomakina, 2014§ |
| 54 | KF801462.2 | Moldova | 7 | Pluta, 2017 [13] |
| 55 | KF801463.2 | Moldova | 7 | Pluta, 2017 [13] |
| 56 | AF399703.3 | Brazil | 1 | Camargos, 2002 [15] |
| 57 | HM563774.3 | Poland | 4 | Rola-Luszczak, 2013 [14] |
| 58 | AF547184.2 | Brazil | 1 | Camargos, 2007 [16] |
| 59 | AY185360.2 | Brazil | 6 | Camargos, 2004 § |
| 60 | AF399704.3 | Brazil | 2 | Camargos, 2002 [15] |
| 61 | AY151262.2 | Brazil | 1 | Stancek, 2004§ |
| 62 | FJ808571.1 | Argentina | 1 | Rodriguez, 2009 [17] |
| 63 | FJ808572.1 | Argentina | 1 | Rodriguez, 2009 [17] |
| 64 | FJ808573.1 | Argentina | 1 | Rodriguez, 2009 [17] |
| 65 | FJ808574.1 | Argentina | 2 | Rodriguez, 2009 [17] |
| 66 | FJ808576.1 | Argentina | 1 | Rodriguez, 2009 [17] |
| 67 | FJ808577.1 | Argentina | 2 | Rodriguez, 2009 [17] |
| 68 | FJ808578.1 | Argentina | 1 | Rodriguez, 2009 [17] |
| 69 | FJ808579.1 | Argentina | 2 | Rodriguez, 2009 [17] |
| 70 | EU262555.2 | Poland | 7 | Pluta, 2017 [13] |
| 71 | EU262575.2 | Poland | 4 | Pluta, 2017 [13] |
| 72 | EF065638.1 | Belgium | 4 | Zhao, 2007 [18] |
| 73 | EF065645.1 | Costa Rica | 5 | Zhao, 2007 [18] |
| 74 | EF065640.1 | Costa Rica | 1 | Zhao, 2007 [18] |
| 75 | EF065635.1 | Costa Rica | 5 | Zhao, 2007 [18] |
| 76 | EF065637.1 | Costa Rica | 1 | Zhao, 2007 [18] |
| 77 | EF065639.1 | Costa Rica | 5 | Zhao, 2007 [18] |
| 78 | EF065655.1 | Costa Rica | 5 | Zhao, 2007 [18] |
| 79 | EF065654.1 | Costa Rica | 5 | Zhao, 2007 [18] |
| 80 | EF065643.1 | Costa Rica | 5 | Zhao, 2007 [18] |
| 81 | S83530.1 | Italy | 7 | Molteni, 1996 [19] |
| 82 | EU266061.1 | Iran | 1 | Hemmatzadeh, 2007 [20] |
| 83 | EF065657.1 | Japan | 1 | Zhao, 2007 [18] |
| 84 | EF065651.1 | Japan | 1 | Zhao, 2007 [18] |
| 85 | EF065653.1 | Japan | 1 | Zhao, 2007 [18] |
| 86 | EF065650.1 | Japan | 3 | Zhao, 2007 [18] |
| 87 | EF065646.1 | Japan | 1 | Zhao, 2007 [18] |
| 88 | EF065658.1 | Japan | 1 | Zhao, 2007 [18] |
| 89 | EF065659.1 | Japan | 1 | Zhao, 2007 [18] |
| 90 | EF065660.1 | Japan | 1 | Zhao, 2007 [18] |
| 91 | EF065661.1 | Japan | 1 | Zhao, 2007 [18] |
| 92 | EF065662.1 | Japan | 1 | Zhao, 2007 [18] |
| 93 | JQ686096.1 | Russia | 4 | Lomakina, 2013§ |
| 94 | JQ686093.1 | Russia | 4 | Lomakina, 2013§ |
| 95 | JQ686094.1 | Russia | 4 | Lomakina, 2013§ |
| 96 | JQ686095.1 | Russia | 4 | Lomakina, 2013§ |
| 97 | FJ808592.1 | Argentina | 2 | Rodriguez, 2009 [17] |
| 98 | FJ808593.1 | Argentina | 2 | Rodriguez, 2009 [17] |
| 99 | FJ808594.1 | Argentina | 2 | Rodriguez, 2009 [17] |
| 100 | FJ808595.1 | Argentina | 4 | Rodriguez, 2009 [17] |
| 101 | FJ808597.1 | Argentina | 2 | Rodriguez, 2009 [17] |
| 102 | FJ808598.1 | Argentina | 2 | Rodriguez, 2009 [17] |
| 103 | JQ686089.1 | Russia | 4 | Lomakina, 2014§ |
| 104 | JQ686091.1 | Russia | 4 | Lomakina, 2014§ |
| 105 | JQ686090.1 | Russia | 4 | Lomakina, 2014§ |
| 106 | JQ686092.1 | Russia | 4 | Lomakina, 2014§ |
| 107 | JQ675757.1 | Russia | 7 | Lomakina, 2013§ |
| 108 | JQ675759.1 | Russia | 8 | Lomakina, 2013§ |
| 109 | JQ675760.1 | Russia | 8 | Lomakina, 2013§ |
| 110 | JQ675756.1 | Russia | 7 | Lomakina, 2013§ |
| 111 | JQ675758.1 | Russia | 4 | Lomakina, 2013§ |
| 112 | JQ686098.1 | Russia | 4 | Lomakina, 2013§ |
| 113 | JQ686118.1 | Russia | 4 | Lomakina, 2013§ |
| 114 | JQ686117.1 | Russia | 4 | Lomakina, 2013§ |
| 115 | JQ686119.1 | Russia | 7 | Lomakina, 2013§ |
| 116 | JQ686120.1 | Russia | 7 | Lomakina, 2013§ |
| 117 | JQ686112.1 | Russia | 4 | Lomakina, 2013§ |
| 118 | JQ686111.1 | Russia | 4 | Lomakina, 2013§ |
| 119 | JQ686116.1 | Russia | 7 | Lomakina, 2013§ |
| 120 | FJ808591.1 | Argentina | 2 | Rodriguez, 2009 [17] |
| 121 | FJ808580.1 | Argentina | 2 | Rodriguez, 2009 [17] |
| 122 | FJ808581.1 | Argentina | 2 | Rodriguez, 2009 [17] |
| 123 | FJ808582.1 | Argentina | 6 | Rodriguez, 2009 [17] |
| 124 | FJ808583.1 | Argentina | 2 | Rodriguez, 2009 [17] |
| 125 | FJ808584.1 | Argentina | 1 | Rodriguez, 2009 [17] |
| 126 | FJ808586.1 | Argentina | 1 | Rodriguez, 2009 [17] |
| 127 | FJ808587.1 | Argentina | 2 | Rodriguez, 2009 [17] |
| 128 | FJ808588.1 | Argentina | 1 | Rodriguez, 2009 [17] |
| 129 | FJ808589.1 | Argentina | 1 | Rodriguez, 2009 [17] |
| 130 | FJ808590.1 | Argentina | 2 | Rodriguez, 2009 [17] |
| 131 | JQ686106.1 | Russsia | 4 | Lomakina, 2013§ |
| 132 | JQ686107.1 | Russia | 4 | Lomakina, 2013§ |
| 133 | EU266063.1 | Iran | 1 | Hemmatzadeh, 2007 [20] |
| 134 | EF065647.1 | USA | 3 | Zhao, 2007 [18] |
| 135 | EF065648.1 | USA | 3 | Zhao, 2007 [18] |
| 136 | EF065649.1 | USA | 3 | Zhao, 2007 [18] |
| 137 | EF065644.1 | USA | 1 | Zhao, 2007 [18] |
| 138 | EF065641.1 | USA | 1 | Zhao, 2007 [18] |
| 139 | EF065642.1 | USA | 1 | Zhao, 2007 [18] |
| 140 | AF111171.1 | Poland | 4 | Reichert, 2016§ |
| 141 | LC080651.1 | Paraguay | 1 | Polat, 2016 [21] |
| 142 | LC080652.1 | Paraguay | 1 | Polat, 2016 [21] |
| 143 | LC154848.1 | Myanmar | 10 | Polat, 2017 [8] |
| 144 | LC080654.1 | Peru | 2 | Polat, 2016 [21] |
| 145 | LC080659.1 | Bolivia | 9 | Polat, 2016 [21] |
| 146 | LC080660.1 | Bolivia | 9 | Polat, 2016 [21] |
| 147 | LC080661.1 | Bolivia | 9 | Polat, 2016 [21] |
| 148 | LC080662.1 | Bolivia | 9 | Polat, 2016 [21] |
| 149 | LC080663.1 | Bolivia | 9 | Polat, 2016 [21] |
| 150 | LC080653.1 | Paraguay | 1 | Polat, 2016 [21] |
| 151 | LC080655.1 | Paraguay | 2 | Polat, 2016 [21] |
| 152 | LC080656.1 | Paraguay | 6 | Polat, 2016 [21] |
| 153 | LC080657.1 | Paraguay | 6 | Polat, 2016 [21] |
| 154 | LC080658.1 | Paraguay | 6 | Polat, 2016 [21] |
| 155 | LC080664.1 | Bolivia | 9 | Polat, 2016 [21] |
| 156 | LC080665.1 | Bolivia | 9 | Polat, 2016 [21] |
| 157 | LC080666.1 | Bolivia | 9 | Polat, 2016 [21] |
| 158 | LC080667.1 | Bolivia | 9 | Polat, 2016 [21] |
| 159 | LC080669.1 | Bolivia | 9 | Polat, 2016 [21] |
| 160 | LC080670.1 | Bolivia | 9 | Polat, 2016 [21] |
| 161 | LC080671.1 | Bolivia | 9 | Polat, 2016 [21] |
| 162 | LC080672.1 | Bolivia | 9 | Polat, 2016 [21] |
| 163 | LC080673.1 | Bolivia | 9 | Polat, 2016 [21] |
| 164 | LC080674.1 | Bolivia | 9 | Polat, 2016 [21] |
| 165 | LC080675.1 | Bolivia | 9 | Polat, 2016 [21] |
| 166 | LC154849.1 | Myanmar | 10 | Polat, 2017 [8] |
| 167 | LC007992.1 | Japan | 1 | Mekata, 2014§ |
| 168 | LC007983.1 | Japan | 1 | Mekata, 2014§ |
| 169 | LC007977.1 | Japan | 1 | Mekata, 2014§ |
| 170 | LC007982.1 | Japan | 1 | Mekata, 2014§ |
| 171 | LC007979.1 | Japan | 1 | Mekata, 2014§ |
| 172 | LC007987.1 | Japan | 1 | Mekata, 2014§ |
| 173 | LC007993.1 | Japan | 3 | Mekata, 2014§ |
| 174 | LC007980.1 | Japan | 1 | Mekata, 2014§ |
| 175 | LC007989.1 | Japan | 1 | Mekata, 2014§ |
| 176 | LC007985.1 | Japan | 1 | Mekata, 2014§ |
| 177 | LC007988.1 | Japan | 1 | Mekata, 2014§ |
| 178 | LC007990.1 | Japan | 1 | Mekata, 2014§ |
| 179 | LC007986.1 | Japan | 1 | Mekata, 2014§ |
| 180 | LC007984.1 | Japan | 1 | Mekata, 2014§ |
| 181 | LC007981.1 | Japan | 1 | Mekata, 2014§ |
| 182 | LC007991.1 | Japan | 1 | Mekata, 2014§ |
| 183 | LC007978.1 | Japan | 1 | Mekata, 2014§ |
| 184 | FM209472.1 | Uruguay | 1 | Moratorio, 2010 [22] |
| 185 | FM209470.1 | Uruguay | 1 | Moratorio, 2010 [22] |
| 186 | FM209469.1 | Uruguay | 1 | Moratorio, 2010 [22] |
| 187 | FM209468.1 | Uruguay | 1 | Moratorio, 2010 [22] |
| 188 | FJ914764.1 | Argentina | 2 | Dube, 2009 [23] |
| 189 | KU233527.1 | Thailand | 10 | Lee, 2016 [24] |
| 190 | KU233528.1 | Thailand | 1 | Lee, 2016 [24] |
| 191 | KU233529.1 | Thailand | 1 | Lee, 2016 [24] |
| 192 | KU233530.1 | Thailand | 6 | Lee, 2016 [24] |
| 193 | KU233531.1 | Thailand | 6 | Lee, 2016 [24] |
| 194 | KP201466.1 | South Korea | 1 | Lee, 2015 [25] |
| 195 | KP201463.1 | South Korea | 1 | Lee, 2015 [25] |
| 196 | KP201460.1 | South Korea | 1 | Lee, 2015 [25] |
| 197 | KP201468.1 | South Korea | 1 | Lee, 2015 [25] |
| 198 | KP201471.1 | South Korea | 1 | Lee, 2015 [25] |
| 199 | KP201461.1 | South Korea | 1 | Lee, 2015 [25] |
| 200 | KP201462.1 | South Korea | 1 | Lee, 2015 [25] |
| 201 | KP201469.1 | South Korea | 1 | Lee, 2015 [25] |
| 202 | KP201470.1 | South Korea | 1 | Lee, 2015 [25] |
| 203 | KP201467.1 | South Korea | 1 | Lee, 2015 [25] |
| 204 | KP201464.1 | South Korea | 3 | Lee, 2015 [25] |
| 205 | KP201465.1 | South Korea | 3 | Lee, 2015 [25] |
| 206 | KP201474.1 | South Korea | 1 | Lee, 2015 [25] |
| 207 | KP201472.1 | South Korea | 1 | Lee, 2015 [25] |
| 208 | KP201480.1 | South Korea | 1 | Lee, 2015 [25] |
| 209 | KP201476.1 | South Korea | 1 | Lee, 2015 [25] |
| 210 | KP201477.1 | South Korea | 1 | Lee, 2015 [25] |
| 211 | KP201478.1 | South Korea | 1 | Lee, 2015 [25] |
| 212 | KP201473.1 | South Korea | 1 | Lee, 2015 [25] |
| 213 | KP201481.1 | South Korea | 1 | Lee, 2015 [25] |
| 214 | KP201479.1 | South Korea | 1 | Lee, 2015 [25] |
| 215 | KP201475.1 | South Korea | 1 | Lee, 2015 [25] |
| 216 | KP201482.1 | South Korea | 1 | Lee, 2015 [25] |
| 217 | KU233532.1 | Thailand | 1 | Lee, 2016 [24] |
| 218 | KU233533.1 | Thailand | 1 | Lee, 2016 [24] |
| 219 | KU233534.1 | Thailand | 10 | Lee, 2016 [24] |
| 220 | KU233535.1 | Thailand | 10 | Lee, 2016 [24] |
| 221 | KU233536.1 | Thailand | 6 | Lee, 2016 [24] |
| 222 | KU233537.1 | Thailand | 10 | Lee, 2016 [24] |
| 223 | KU233538.1 | Thailand | 10 | Lee, 2016 [24] |
| 224 | KU233539.1 | Thailand | 10 | Lee, 2016 [24] |
| 225 | KU233540.1 | Thailand | 10 | Lee, 2016 [24] |
| 226 | KU233541.1 | Thailand | 10 | Lee, 2016 [24] |
| 227 | KU233542.1 | Thailand | 10 | Lee, 2016 [24] |
| 228 | KU233543.1 | Thailand | 10 | Lee, 2016 [24] |
| 229 | KU233544.1 | Thailand | 6 | Lee, 2016 [24] |
| 230 | KU233545.1 | Thailand | 10 | Lee, 2016 [24] |
| 231 | KU233546.1 | Thailand | 10 | Lee, 2016 [24] |
| 232 | KU233547.1 | Thailand | 10 | Lee, 2016 [24] |
| 233 | KU233548.1 | Thailand | 6 | Lee, 2016 [24] |
| 234 | KU233549.1 | Thailand | 6 | Lee, 2016 [24] |
| 235 | KU233550.1 | Thailand | 10 | Lee, 2016 [24] |
| 236 | KU233551.1 | Thailand | 10 | Lee, 2016 [24] |
| 237 | KU233552.1 | Thailand | 10 | Lee, 2016 [24] |
| 238 | KU233553.1 | Thailand | 10 | Lee, 2016 [24] |
| 239 | KU233554.1 | Thailand | 10 | Lee, 2016 [24] |
| 240 | KU233555.1 | Thailand | 10 | Lee, 2016 [24] |
| 241 | KU233556.1 | Thailand | 1 | Lee, 2016 [24] |
| 242 | KU233557.1 | Thailand | 1 | Lee, 2016 [24] |
| 243 | KU233558.1 | Thailand | 1 | Lee, 2016 [24] |
| 244 | KU233559.1 | Thailand | 1 | Lee, 2016 [24] |
| 245 | KU233560.1 | Thailand | 1 | Lee, 2016 [24] |
| 246 | KU233561.1 | Thailand | 10 | Lee, 2016 [24] |
| 247 | KU233562.1 | Thailand | 6 | Lee, 2016 [24] |
| 248 | KU233563.1 | Thailand | 6 | Lee, 2016 [24] |
| 249 | KU233564.1 | Thailand | 1 | Lee, 2016 [24] |
| 250 | KU233565.1 | Thailand | 1 | Lee, 2016 [24] |
| 251 | KU233566.1 | Thailand | 1 | Lee, 2016 [24] |
| 252 | KU233567.1 | Thailand | 1 | Lee, 2016 [24] |
| 253 | AF503581 | Belgium | 4 | Willems, 1993 [26] |
| 254 | KX674372.1 | Caribbean | 1 | Yang, 2016 [27] |
| 255 | KT122858.1 | Belgium | 4 | Rosewick, 2015 [28] |
| 256 | KX674368.1 | Caribbean | 1 | Yang, 2016 [29] |

§- direct submission to GenBank

**References:**

1. Sagata N, Yasunaga T, Tsuzuku-Kawamura J, Ohishi K, Ogawa Y, Ikawa Y. Complete nucleotide sequence of the genome of bovine leukemia virus: its evolutionary relationship to other retroviruses. Proc Natl Acad Sci U S A. 1985;82(3):677-81
2. Dube S, Dolcini G, Abbott L, Mehta S, Dube D, Gutierrez S, Ceriani C, Esteban E, Ferrer J, Poiesz B. The complete genomic sequence of a BLV strain from a Holstein cow from Argentina. Virology. 2000;277(2):379-86.
3. Moratorio G, Fischer S, Bianchi S, Tomé L, Rama G, Obal G, Carrión F, Pritsch O, Cristina J. A detailed molecular analysis of complete Bovine Leukemia Virus genomes isolated from B-cell lymphosarcomas. Vet Res. 2013;44(1):19.
4. Mamoun RZ, Morisson M, Rebeyrotte N, Busetta B, Couez D. Sequence variability of bovine leukemia virus env gene and its relevance to the structure and antigenicity of the glycoproteins. J Virol. 1990;64:4180–4188.
5. Rice NR, Stephens RM, Couez D, Deschamps J, Kettmann R, Burny A, Gilden RV. The nucleotide sequence of the env gene and post-env region of bovine leukemia virus. Virology. 1984;138(1):82-93.
6. Petropoulos CJ. Retroviral Taxonomy, Protein Structure, Sequences, and Genetic Maps. Retroviruses. Coffin, JM, editor. Cold Spring Harbor: Cold Spring Harbor Laboratory, 1997.
7. Mekata H, Sekiguchi S, Konnai S, Kirino Y, Horii Y, Norimine J. Horizontal transmission and phylogenetic analysis of bovine leukemia virus in two districts of Miyazaki, Japan. J Vet Med Sci. 2015;77(9):1115-20.
8. Polat M, Moe HH, Shimogiri T, Moe KK, Takeshima SN, Aida Y. The molecular epidemiological study of bovine leukemia virus infection in Myanmar cattle. Arch Virol. 2017;162(2):425-437.
9. Murakami H, Uchiyama J, Nikaido S, Sato R, Sakaguchi M, Tsukamoto K. Inefficient viral replication of bovine leukemia virus induced by spontaneous deletion mutation in the G4 gene. J Gen Virol. 2016;97(10):2753-2762.
10. Limansky AP, Limanskaya OY. Comparison of primer sets for the detection of bovine leukemia virus by polymerase chain reaction. Bull. Vet. Inst. Pulawy. 2002;46:27-36.
11. Lee AJ, Kim EJ, Joung HK, Kim BH, Song JY, Cho IS, Lee KK, and Yeun-Kyung Shin YK. Sequencing and phylogenetic analysis of the gp51 gene from Korean bovine leukemia virus isolates. Virol J. 2015; doi: 10.1186/s12985-015-0286-4.
12. Coulston J, Naif H, Brandon R, Kumar S, Khan S, Daniel RCW, Lavin MF. Molecular cloning and sequencing of an Australian isolate of proviral bovine leukemia virus DNA: comparison with other isolates. Journal of General Virology. 1990;71:1737-1746.
13. Pluta A, Rola-Łuszczak M, Kubiś P, Balov S, Moskalik R, Choudhury B, Kuźmak J. Molecular characterization of bovine leukemia virus from Moldovan dairy cattle. Arch Virol. 2017; doi: 10.1007/s00705-017-3241-4.
14. Rola-Łuszczak M, Pluta A, Olech M, Donnik I, Petropavlovskiy M, Gerilovych A, Vinogradova I, Choudhury B, Kuźmak J. The molecular characterization of bovine leukaemia virus isolates from Eastern Europe and Siberia and its impact on phylogeny. PLoS One. 2013; doi: 10.1371/journal.pone.0058705.
15. Camargos MF, Stancek D, Rocha MA, Lessa LM, Reis JK. Partial sequencing of env gene of bovine leukaemia virus from Brazilian samples and phylogenetic analysis. J Vet Med B Infect Dis Vet Public Health. 2002; doi:10.1046/j.1439-0450.2002.00582.x.
16. Camargos MF, Pereda A, Stancek D, Rocha MA, dos Reis JK, Greiser-Wilke I, Leite RC.Molecular characterization of the env gene from Brazilian field isolates of Bovine leukemia virus. Virus Genes. 2007;34(3):343-50.
17. Rodriguez SM, Golemba MD, Campos RH, Trono K, Jones LR. Bovine leukemia virus can be classified into seven genotypes: evidence for the existence of two novel clades. J Gen Virol. 2009;90:2788–2797.
18. Zhao X, Buehring GC. Natural genetic variations in bovine leukemia virus envelope gene: possible effects of selection and escape. Virology. 2007;366:150–165.
19. Molteni E, Agresti A, Meneveri R, Marozzi A, Malcovati M, Bonizzi L, Poli G, Ginelli E. Molecular characterization of a variant of proviral bovine leukaemia virus (BLV). Zentralbl. Veterinarmed. Beih.1996;p. 201-211.
20. Hemmatzadeh F. Sequencing and phylogenetic analysis of gp51 gene of bovine leukaemia virus in Iranian isolates. Vet Res Commun. 2007;31(6):783–789.
21. Polat M, Takeshima SN, Hosomichi K, Kim J, Miyasaka T, Yamada K, Arainga M, Murakami T, Matsumoto Y, Barra Diaz V, Panei CJ, González ET, Kanemaki M, Onuma M, Giovambattista G, Aida Y. A new genotype of bovine leukemia virus in South America identified by NGS-based whole genome sequencing and molecular evolutionary genetic analysis. Retrovirology. 2016;13(1):4.
22. Moratorio G, Obal G, Dubra A, Correa A, Bianchi S, Buschiazzo A, Cristina J, Pritsch O. Phylogenetic analysis of bovine leukemia viruses isolated in South America reveals diversification in seven distinct genotypes. Arch Virol. 2010;155(4):481-9.
23. Dube S, Abbott L, Dube DK, Dolcini G, Gutierrez S, Ceriani C, Juliarena M, Ferrer J, Perzova R, Poiesz BJ. The complete genomic sequence of an in vivo low replicating BLV strain. Virol J. 2009;6:120.
24. Lee E, Kim EJ, Ratthanophart J, Vitoonpong R, Kim BH, Cho IS, Song JY, Lee KK, Shin YK. Molecular epidemiological and serological studies of bovine leukemia virus (BLV) infection in Thailand cattle. Infect Genet Evol. 2016;41:245–254.
25. Lee AJ, Kim EJ, Joung HK, Kim BH, Song JY, Cho IS, Lee KK, and Yeun-Kyung Shin YK. Sequencing and phylogenetic analysis of the gp51 gene from Korean bovine leukemia virus isolates. Virol J. 2015; doi: 10.1186/s12985-015-0286-4.
26. Willems L, Thienpont E, Kerkhofs P, Burny A, Mammerickx M, Kettmann R. Bovine leukemia virus an animal model for the study of intrastrain variability. J Virol. 1993;67:1086–1089.
27. Yang Y, Kelly PJ, Bai J, Zhang R, Wang Ch. First Molecular Characterization of Bovine Leukemia Virus Infections in the Caribbean. PLoS One. 2016;11(12):e0168379.
28. Rosewick N, Durkin K, Artesi M, Marçais A, Hahaut V, Griebel P, Arsic N, Avettand-Fenoel V, Burny A, Charlier C, Hermine O, Georges M, Van den Broeke A. Cis-perturbation of cancer drivers by the HTLV-1/BLV proviruses is an early determinant of leukemogenesis. Nat Commun. 2017; doi: 10.1038/ncomms15264.
29. Yang Y, Kelly PJ, Bai J, Zhang R, Wang Ch. First Molecular Characterization of Bovine Leukemia Virus Infections in the Caribbean. PLoS One. 2016;11(12):e0168379.
