# Supplementary material for: Computational analysis of envelope glycoproteins from diverse geographical isolates of bovine leukemia virus identifies highly conserved peptide motifs
Source: Retrovirology. 2018 Jan 8;15:2. doi: 10.1186/s12977-017-0383-0 (PMC5759284; doi:10.1186/s12977-017-0383-0)
Supplement: Supplementary file 2 — Additional file 2: Table S2. The conserved and semi-conserved segments in BLV envelope glycoprotein. A white color reflect segments highly conserved at the amino and nucleotide sequence levels, dark yellow color reflect segments conserved at the amino acid level. # The sequences are from MEME output and they reflect the nucleotides that are most frequent at these positions. For example, having a T in the output sequence does not mean that all gp51 env segments of BLVs will have a T nucleotide at that position. A [|] indicates more than one nucleotide is frequent at this position. A “-“ horizontal dashes between nucleotides denote the next codon. *The amino acid residues that are most frequent at these positions. [file 12977_2017_383_MOESM2_ESM.docx]

**Spplementary Table 2.** The conserved and semi-conserved MEME motifs in BLV envelope glycoprotein.

| MEME motif | MEME Motifs Nucleotide Sequence# | Prevalent Amino Acid Sequence* |
| --- | --- | --- |
| A | GGA-GCC[A]-AG[A]G-GCC[A]-A[G]TG-G[A]T[C]C[TǀG]-A[G]CA[G]-T[C]AT-GAT[C]-TGC-GAG[A]-CCC-CGA-TGC-CCT[C]-TAT[C]-G[A]TG-GGG[A]-GCA[GǀCǀT] | GARAMVTYDCEPRCPYVGA |
| B | GAC[T]-TGG-G[A]TT[C]-CCC-T[C]CT-G[A]TC-AG[A]A[G]-TCA[C]-TGG-GCC[T]-CTG[A] | DWVPSVRSWAL |
| C | ACC-CAA[G]-G[A]GA[G]-TGG-C[G]AC-CAC-CC[T]T-TCC-CAG-AG[A]G[A]-TTG[A]-TTG[A] | TQGWHHPSQRLL |
| D | C[G]TC[T]-AA[G]A-CA[G]A-T[C]GT[C]-CAT[C]-GGA-A[G]TT[C]-T[C]TC | LKQCHGIF |
| E | TTC-G[A]AC-TGC[T]-CCC-CAC-TGG-GA[G]C-AAT[C]-GCC[T] | FDCPHWDNA |
| F | ATA-T[A]G[A]G-GGA-TAT-GAT[C]-CCC[T]-C[TA]TG[A]-ATC[T]-ACC[GǀT]-TTT[C]-TCT[C]-TTA | IWGYDPLITFSL |
| G | CA[G]A[G]-TC[T]A[G]-CCT[C]-TTC[T]-TGT[C]-G[C]C[T]C-AA[G]G-TCT-CCC[TǀG]-A[C]GA[G]-TAC[T]-ACC[T] | QSPFCAKSPRYT |
| H | G[A]T[G]T-AAT-CTC[T]-TC[T]-ACG[A]-G[A]C[TǀG]T[C]-TC[T]C-TC[T]C[T]-GC[T]C-CCT-C[T]C[T]T[C]-ACC-CGG-GT[C]C[G]-A[C]G[A]A[G]-C[G]G[C]T[CǀG] | VNLSTASSAPPTRVRR |
| I | T[C]T[C]G[A]-GAC[T]-TT[C]T[CǀG]-G[A]TA[CǀG]-AA[G]T[GǀC]-G[A]GC[TǀG]-TAT[C]-CCT[C]-AAG[A]-ATC[T]-TAC[T]-TGG-CCC[G] | LDFVNGYPKIYWP |
| J | CA[G]G[A]-G[AǀC]C[TǀA]C[T]-G[A]AT-CA[G]A[G]-GGA[TǀG]-TC[T]C-TTT-T[C]AT-G[A]T[C]C[T]-A[GǀC]A[G]T-C[T]A[G]T[C]-CA[G]G-AT[C]T[C] | QADQGSFYVNHQI |
| K | ATG-A[GǀC]C[T]A-G[A]C[T]A-T[C]AT[C]-A[C]AC[T]-CA[T]A[TǀG] | MTAYNQ |
